# Improving farming practices reduces the carbon footprint of spring wheat production

**DOI:** 10.1038/ncomms6012

**Published:** 2014-11-18

**Authors:** Yantai Gan, Chang Liang, Qiang Chai, Reynald L. Lemke, Con A. Campbell, Robert P. Zentner

**Affiliations:** 1 Gansu Provincial Key Laboratory for Aridland Crop Science, Gansu Agricultural University, Lanzhou 730070, China; 2 Semiarid Prairie Agricultural Research Centre, Agriculture and Agri-Food Canada, Swift Current, Saskatchewan, Canada S9H 3X2; 3 Pollutant Inventories and Reporting Division, Environment Canada, 9th floor, Fontaine Building, 200 Sacré-Coeur, Gatineau, Québec, Canada K1A 0H3; 4 Saskatoon Research Centre, Agriculture and Agri-Food Canada, Saskatoon, Saskatchewan, Canada S7N 0X2; 5 Eastern Cereal and Oilseed Research Centre, Agriculture and Agri-Food Canada, 960 Carling Avenue, Ottawa, Ontario, Canada K1A 0C6

## Abstract

Wheat is one of the world’s most favoured food sources, reaching millions of people on a daily basis. However, its production has climatic consequences. Fuel, inorganic fertilizers and pesticides used in wheat production emit greenhouse gases that can contribute negatively to climate change. It is unknown whether adopting alternative farming practices will increase crop yield while reducing carbon emissions. Here we quantify the carbon footprint of alternative wheat production systems suited to semiarid environments. We find that integrating improved farming practices (that is, fertilizing crops based on soil tests, reducing summerfallow frequencies and rotating cereals with grain legumes) lowers wheat carbon footprint effectively, averaging −256 kg CO_2_ eq ha^−1^ per year. For each kg of wheat grain produced, a net 0.027–0.377 kg CO_2_ eq is sequestered into the soil. With the suite of improved farming practices, wheat takes up more CO_2_ from the atmosphere than is actually emitted during its production.

## Abstract (web summary)

Wheat is the world’s second most favoured food source, yet its production has climatic consequences. Here the authors quantify the carbon footprint of wheat production systems and evaluate potential alternative farming practices, with a reduced negative impact on climate.

## Graphical abstract

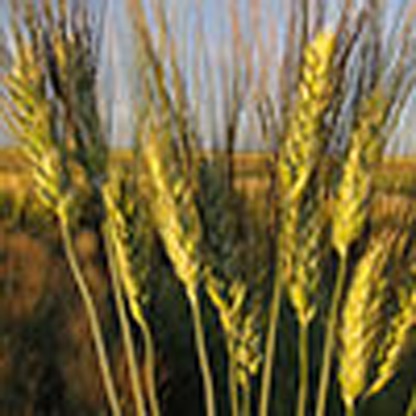

Global demands for major grains such as wheat (*Triticum aestivum* L.) are projected to increase by 70% by 2050 (ref. 1), driven by the ever-growing human population’s need for food, feed, fibre and fuel^2^,^3^. To meet this target, grain production must increase substantially, while, at the same time, agriculture,s environmental footprint must shrink dramatically^4^. Given the limited availability of uncultivated land on the planet^5^ and the growing environmental concerns related to converting carbon-rich forests and grasslands to cropland^3^, the future increase in grain production must mostly come from existing farmland^6^.

Wheat is the third largest cereal crop (after maize—*Zea Mays*, and rice—*Oryza sativa*) grown on the planet^7^. In semiarid rain-fed areas, wheat is traditionally grown in a wheat-fallow or wheat-wheat-fallow cropping system^8^. In the fallow year, the land is left unplanted for the entire growing season and multiple operations of tillage are used to control weeds^9^. This farming practice has been used in many arid and semiarid regions of the world, such as Australia^10^, northwest China^11^, northwest Eurasia^12^, central Africa^13^ and the North American prairies. It is believed that summerfallowing allows a large proportion of rainfall to be conserved in the soil profile, which is available for the crops grown in the following year^14^. Also, the soils in the fallow period release nitrogen (N) via N mineralization of soil organic matter^15^,^16^, benefiting the crops grown in subsequent years^17^. However, recent evidence has shown that summerfallowing can result in serious environmental consequences^18^,^19^, as more fossil fuel is required for the multiple tillage operations during the fallow period^19^. Also, soil organic matter can be depleted with a higher frequency of fallowing^20^, causing the degradation of soil quality and increased soil erosion^18^.

The adoption of more intensified cropping systems (for example, reducing the frequency of summerfallow, including legumes in the rotation, use of higher inputs of inorganic fertilizers and chemicals) has been shown to increase crop yields compared with traditional fallow-wheat or wheat monoculture systems^21^. However, the increased use of inorganic fertilizers and pesticides in high-yielding systems increases greenhouse gas emissions^22^,^23^. Also, there is growing evidence of yield plateaus or abrupt decreases in the rate of yield gain over years^24^. In some areas, crop yields have either stagnated or even collapsed in recent years^25^. This evidence clearly indicates that the high-yielding systems can also have negative climate consequences. It is unclear whether some individual farming practices that have been proven to be essential and successful in crop production can be packaged together in a systematic manner to empower the increase of crop yields, while, at the same time, reducing the potentially negative impacts on the environment.

Here we identify the following four key farming practices and integrate them systematically into alternative wheat production systems: (i) inclusion of grain legumes in rotations to fix atmospheric N_2_ into plant-available N and to replace a portion of inorganic N fertilizer in wheat production; (ii) use of annual soil tests to determine soil residual nutrients and potential mineralized N from soil organic matter that may be available for crops, (iii) application of a proper rate of inorganic fertilizers to meet the nutrient requirements by crops and to avoid over- or under-fertilization and (iv) adoption of more intensified crop rotation systems (that is, cropping with reduced frequency of summerfallowing) so as to sequester greater amounts of CO_2_ from the atmosphere to offset carbon emissions associated with crop production inputs. We quantify the climate consequences associated with the adoption of a suite of improved farming practices in alternative wheat production systems. Two metrics of carbon footprints are determined: first, the quantity of greenhouse gases emitted per unit of farmland (for the purpose of simplicity, it is defined as per-area carbon footprint), and second, the quantity of greenhouse gases associated with per kg of grain produced (defined as per-yield carbon footprint). Our central hypothesis is that the packaging together of these individually proven farming practices can increase crop yields, enhance net carbon sequestration and lower the carbon footprint of wheat production. We test the hypothesis using a 25-year (1985–2009) field experiment conducted at the Agriculture and Agri-Food Canada Research Centre, Swift Current, Saskatchewan, Canada. We find that spring wheat produced using the suite of improved farming practices has negative (more desirable) carbon footprints, averaging −256 kg CO_2_ eq ha^−1^ per year. For each kg of wheat grain produced, a net 0.027–0.377 kg CO_2_ eq is sequestered into the soil. Among factors, the choice of cropping systems has the highest impact on the carbon footprint of wheat production, with the lentil-wheat (LentW) rotation system having the lowest per-area carbon footprint at −552 kg CO_2_ eq ha^−1^ and the most negative per-yield carbon footprint at −0.377 kg CO_2_ eq per kg of grain. We conclude that wheat can convert more CO_2_ from the atmosphere into soil carbon than is actually emitted during its production if some key farming practices can be identified and integrated together systematically.

## Results

### Carbon footprints of wheat per area and per kg of grain

Spring wheat produced using the suite of farming practices (described above) had negative (more desirable) carbon footprints, both on the basis of per unit of farmland and per kg of grain yield (Table 1). The 25 study years (1985–2009) were categorized into three groups based on water availability: 7 dry years with annual precipitation (Pr) below 210 mm and the ratio of Pr to evapotranspiration (Pr/PE)=0.291; 13 normal years with annual Pr between 211 and 340 mm and Pr/PE=0.495 and 5 wet years with annual Pr between 341 and 420 mm and Pr/PE=0.687. Negative carbon footprint values were consistently attained in each of the three water-availability categories. Average annual per-area carbon footprint was −165 kg CO_2_ eq ha^−1^ in dry years, −323 in normal years and −274 in wet years. The negative carbon footprints indicate that the production of spring wheat on the semiarid North American prairies sequesters more CO_2_ from the atmosphere than is emitted, and that spring wheat produced with improved farming practices can act as a net sink of CO_2_. Four cropping systems were evaluated in this study, namely (i) fallow-flax-wheat (FFlxW), (ii) fallow-wheat-wheat (FWW), (iii) continuous wheat (ContW) and (iv) LentW. Among these, the LentW system had the lowest per-area carbon footprint at −552 kg CO_2_ eq ha^−1^, which was 127% lower than the ContW system, 153% lower than FWW and 790% lower than FFlxW. The largest negative per-area carbon footprint for the LentW system was due to the lower rates of N fertilizer required by the wheat crop in this rotation, as the preceding lentil plants fixed N_2_ from the atmosphere through its biological association with soil microorganisms thereby building up the soil’s N supplying capacity (more discussion below).

Similarly, when calculated on the basis of per kg of grain yield, wheat cropping systems had negative per-yield carbon footprints, averaging −0.223 kg CO_2_ eq per kg of grain in dry years, −0.176 in normal years and −0.129 in wet years (Table 1). These negative per-yield carbon footprint values indicated that for each kg of wheat grain produced a net amount of 0.027–0.377 kg of CO_2_ eq was captured from the atmosphere across all water availability levels. Among the four cropping systems, the LentW rotation was again most favourable with the most negative per-yield carbon footprint at −0.377 kg CO_2_ eq per kg of grain per year, followed by the ContW system at −0.151 and FWW at −0.164, and FFlxW at −0.027 being the least favourable.

To determine the key factors that contribute to the negative carbon footprint and their relative magnitudes in influencing the carbon footprint values, we used analysis of covariance and found that about 82% of the variation in per-area carbon footprint values was due to differences in the cropping systems (Supplementary Table 1). Soil organic carbon was the second largest contributor (4.4%) to the variation of per-area carbon footprint values. In a more or less similar manner, about 61% of the variation in per-yield carbon footprint was due to differences in the cropping systems, and the second largest contributor to the variation (14.6%) was grain yield. Nitrogen fertilization contributed 6.2% to the variation of per-yield carbon footprint, with the rest of the factors contributing little or none. Here we show that the choice of cropping system in which wheat crops are produced has significant climate consequences, and that the key to lowering the carbon footprint of wheat cropping is the development and adoption of cropping systems with individually proven farming practices integrated together.

The analysis of covariance also revealed significant interactions among some of the crop input factors that influenced carbon footprints (Supplementary Table 1); this suggests that the change of one input factor may impact how the other factors affect the carbon footprint values (sensitivity tests described below provide more insights). However, the analysis of covariance across the three water-availability categories showed that the choice of cropping systems, soil carbon change over time, fertilizer N input and grain yield were the four most important factors influencing the carbon footprint values in wheat cropping. Therefore, more in-depth analyses were conducted for these key factors.

### Soil organic carbon and wheat carbon footprints

**Figure 1:**
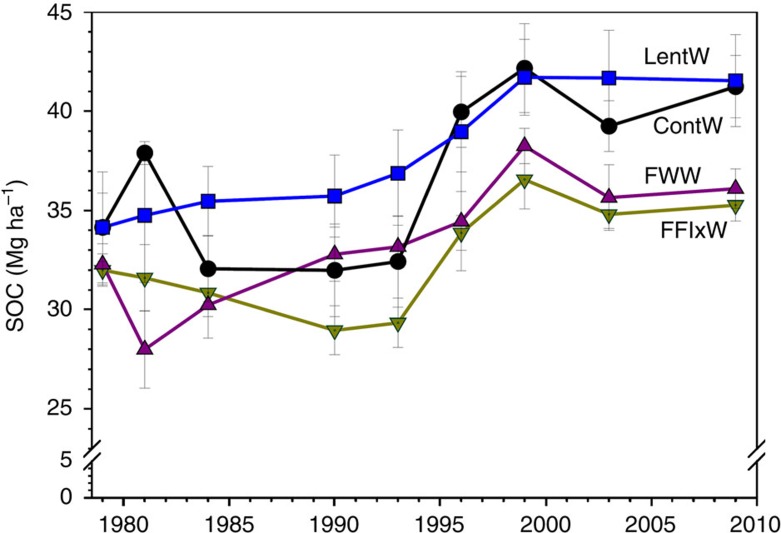
Soil organic carbon in 0–15 cm depth under four cropping systems. The rotation systems are: fallow-flax-wheat (FFlxW), fallow-wheat-wheat (FWW), continuous wheat (ContW) and lentil-wheat (LentW). The line bars are the standard error of the means. Data are for the period 1979–2009.

The base value of soil organic carbon (SOC) measured in 1979 when the LentW system was first introduced was about 33 Mg C ha^−1^. Since then, the SOC under these wheat cropping systems has increased gradually, with the greatest increase occurring between 1993 and 1999 (Fig. 1). The larger gain of SOC during the latter period was due to higher crop productivity^26^ with more biomass carbon returned to the soil^27^. This was accomplished primarily through the higher rates of N fertilizer applied to the crops (with the new soil test recommendation guidelines) coupled with more favourable growing season precipitation in this period. Well-managed cropping systems have been shown to sequester more carbon^28^ with crop residue retention serving as a key factor increasing the quantity of SOC^29^.

**Figure 2:**
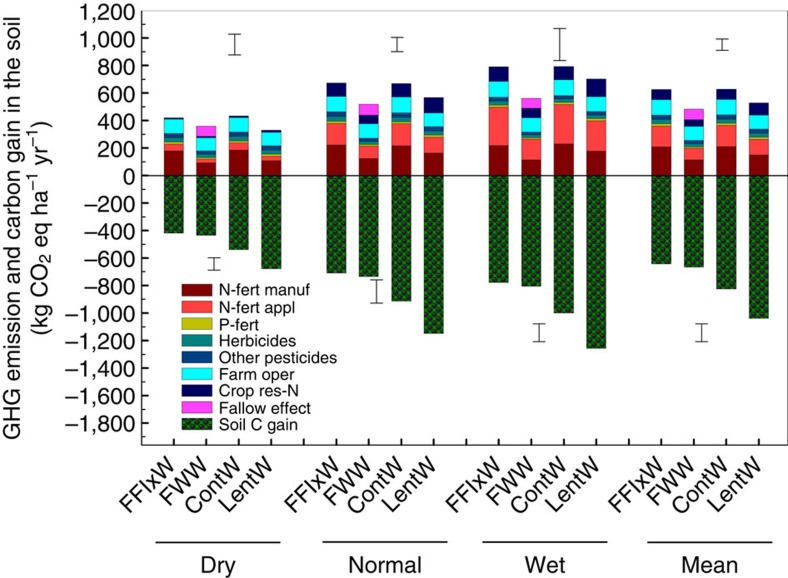
Carbon emissions (top) and sequestrations (bottom) for alternative wheat cropping systems. These rotation systems were tested under dry (the ratio of precipitation to evapotranspiration (Pr/PE)=0.291±0.014), normal (Pr/PE=0.496±0.024) and wet (Pr/PE=0.688±0.036) growing conditions. The line bars are least significant differences (LSDs) with *P*≤0.05 among the four rotation systems within each water-availability category (*n*=3 replicates × 7 years for dry years, 3 × 13 for normal years and 3 × 5 for wet years).

A per unit farmland carbon footprint value represents the balance between carbon emissions and carbon sequestration. Averaged over the 25-year study period, the annual greenhouse gas emissions averaged 357 kg CO_2_ eq ha^−1^ in dry years, 577 in normal years and 687 in wet years (Fig. 2, top portion). The emissions included those from crop residue decomposition, applied inorganic N and phosphorus fertilizers, N leaching losses, application of pesticides, fuel used in various farming operations (such as planting, spraying pesticides, harvesting and so on); and fossil energy used during the manufacture, transportation, storage and delivery of these crop inputs to the farm gate. However, these emissions were more than offset by the greater carbon conversion of wheat plants from atmospheric CO_2_ into plant biomass and ultimately sequestered into the soil (Fig. 2, bottom portion). Consequently, the carbon footprints per unit of farmland became negative (Table 1).

Weather played a significant role in affecting both carbon emissions and carbon sequestration. There was a generally lower carbon emission in drier years because of less production inputs to the crops and lower N_2_O emissions from crop residue decomposition compared with the other years (Fig. 2), whereas in normal and wetter years, more plant biomass carbon was sequestered to the soil. On average, annual soil carbon gain was 877±15 kg CO_2_ eq ha^−1^ in normal years and 961±14 in wet years, which were 69% and 85% more, respectively, than the soil carbon gain obtained in dry years. Greater crop productivity under more favourable weather conditions^27^ leads to greater crop residue and root biomass production^30^, which helps enhance soil organic carbon.

Among the four cropping systems, the LentW system gained an average 1,039 kg CO_2_ eq ha^−1^ annually through soil carbon sequestration, which was 26% more than the gain for ContW, 56% more than for FWW and 62% more than for FFlxW. This cereal–legume rotation had the advantage that the lentil plants fixed N_2_ from the atmosphere^31^, and the increased N availability enhanced plant biomass accumulation^26^. Despite the large variation in carbon emissions and sequestration between the four systems (Fig. 2), the overall ranking of their carbon footprint values was consistent across the dry, normal and wet growing conditions (Table 1). This suggests that spring wheat grown using this suite of improved farming practices can attain a net carbon balance regardless of water availability (except for years with extremely dry weather).

**Figure 3:**
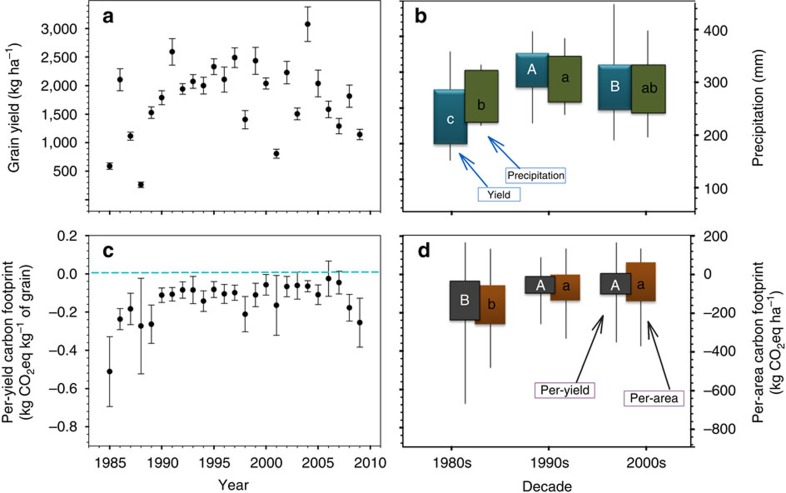
Grain yield and carbon footprints of spring wheat in different years or decades. (**a**) Grain yield in year; (**b**) grain yield and growing-season precipitation in each decade; (**c**) carbon footprint values in each year and (**d**) carbon footprints per-area and per-yield in each decade. The line bars in **a** and **c** are confidence intervals at 95%. In **b** and **d**, the length of the line bars represents the maximum and minimum values, and the height of the boxes represents the upper and lower quartiles. The boxes with uppercase letters denote significant differences (*P*<0.05) between the three decades in (**b**) grain yield or (**d**) per-yield carbon footprint, and the boxes with lowercase letters denote significant differences (*P*<0.05) between the three decades in (**b**) precipitation or (**d**) in per-area carbon footprint.

The SOC level has shown little further change since 1999 (Fig. 1). Coincidentally, crop yields have been in a stagnate state or trending lower since the beginning of the 2000s (Fig. 3a). On average, annual wheat grain yield in the 2000s was 17% lower (*P*<0.01) than the yield obtained in the 1990s (Fig. 3b). Crops received about 10% less precipitation during the growing seasons of the 2000s than in the 1990s. In semiarid areas, water availability is key for crop productivity^32^,^33^.

Calculated by year, the wheat carbon footprint was primarily in the negative territory during the entire 25 study years, and the values in the earlier years were generally more negative (with greater variation) compared with the other years (Fig. 3c). Calculated by decade, the annual mean value of per-area carbon footprint in the 1980s was −181±12 kg CO_2_ eq ha^−1^, significantly lower than those obtained in the 1990s and 2000s (Fig. 3d); similarly, the mean value of per-yield carbon footprint in the 1980s was also lower than those obtained in the 1990s and 2000s. However, the carbon footprint values obtained in the 1990s did not differ from those in the 2000s, even though overall crop yield has been trending lower in recent years (Fig. 3a). In this context, it is clear that the wheat carbon footprint is an outcome of a complex of various factors, including the change in SOC, the quantity and method of crop inputs, the crop yield response and other relevant factors (sensitivity tests below provide more insights).

### The quantity and method of crop inputs and carbon footprints

**Figure 4:**
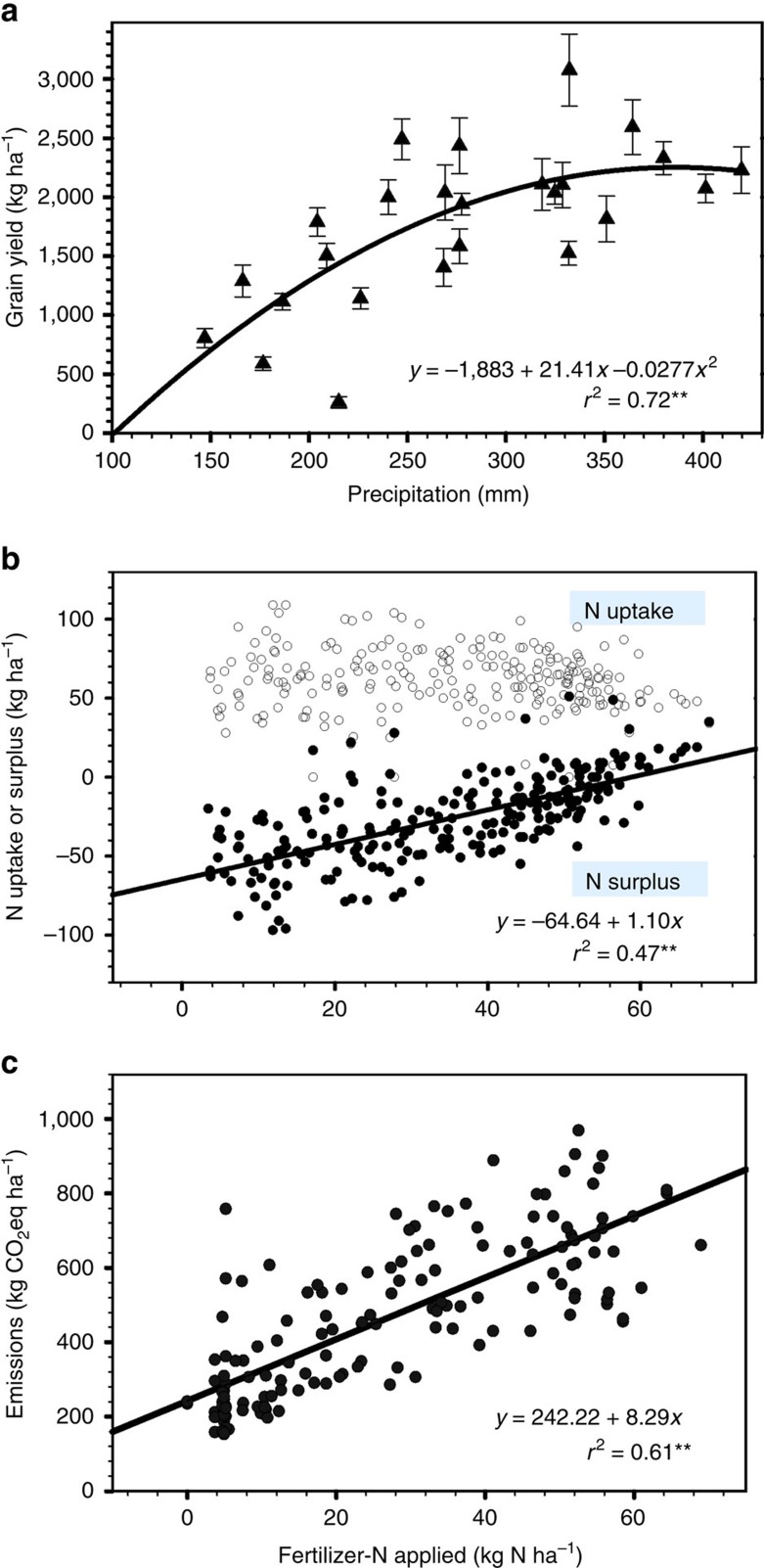
Effects of precipitation and N input on wheat grain yield and carbon emissions. (**a**) Wheat grain yield is a quadratic function of the growing-season precipitation during the 25-year period (the line bars are the standard error of the means); (**b**) increasing fertilizer-N input in wheat production increases the amount of N-surplus in the soil (solid circles) in a linear relationship, whereas N input has no effect on N uptake in the aboveground plant parts (open circles) and (**c**) increasing fertilizer-N input increases carbon emissions and each kg of N increases emissions by 8.29 kg CO_2_ equivalents.

Over the 25-year period, annual wheat grain yield varied from 201 to 3,484 kg ha^−1^ (Table 2), mainly reflecting the level of growing season precipitation, with each millimetre of precipitation increasing grain yield by an average of 21.4 kg ha^−1^ (Fig. 4a). There was a significant interaction between water availability and cropping systems in affecting grain yield. The four systems had a similar level of grain yield in dry years, whereas wheat in the FWW system produced significantly lower grain yields in the normal (24% lower) and wet (27% lower) years than the other systems (Table 2). Summerfallow occupies one-third of the rotation phases in the FWW system, resulting in the low annualized wheat yield over the 3-year rotation phases.

In each of the 25 individual years, wheat was fertilized based on annual soil tests^34^, which varied among the crop rotation systems and between years (Table 2). Among the four cropping systems, FWW received the lowest quantity of N fertilizer because no fertilizer was needed in the fallow phase, whereas the ContW system received the highest quantity of N fertilizer, giving rise to the lowest fertilizer N use efficiency (defined as the kg grain produced per kg of fertilizer-N applied). Nevertheless, the fertilization of wheat crops based on annual soil tests permits a best estimate between N supplies and N requirements by the crops. Consequently, N surplus (defined as N input minus total N uptake by crop plants, with the assumption of soil N state remaining unchanged in a given crop season^35^) was near zero in most years (Fig. 4b). When the above-ground N surplus is equal to or smaller than zero, yield-scaled carbon emissions typically remain small^35^. These results suggest that the amount of N fertilizer applied to the crops in this long-term experiment is adequate in meeting the N requirements for normal plant growth, whereas minimizing carbon emissions associated with N input.

There was a positive relationship between N fertilizer input and carbon emissions, with each kg of N input giving rise to 8.29 kg CO_2_ eq ha^−1^ of emissions (Fig. 4c). This is no surprise as inorganic N fertilizers contributed the greatest percentage of total emissions ascribed to the crop inputs (Supplementary Table 2). On average, 53% of the C emissions came from N sources, of which 22% came from direct N_2_O emissions and indirect N_2_O emissions via volatilization of NH_3_ and NOx and leaching of nitrate from the application of N fertilizers in the field, and the other 31% came from the manufacture, transportation, storage and delivery of N fertilizers to farm gates before farm use. The contribution of N fertilizer to total C emissions was 16.7 times that of P fertilizer, 8.4 times that of pesticides and 2.3 times that of the various on-farm cultural and tillage operations. These numbers are in the range of the values reported from the other semiarid wheat production areas with comparable systems, such as the North American prairies^36^,^37^,^38^, Finland^39^, New South Wales of Australia^40^, the United Kingdom^41^ and the northern China Plains^42^.

A closer examination of the relationship between N supply and N uptake revealed that fertilizer-N contributed a portion of the N in the harvested grain and straw, with the remaining N uptake coming from residual soil N and N mineralized from soil organic matter during the growing season (data not shown). In semiarid climates, soil-sourced N, mostly through the released inorganic N from N mineralization^43^, contributes a large portion of the N uptake by crops^44^. Here we show that use of annual soil tests to quantify N fertilizer requirements of crops is a simple and key practice to ensure a proper nutrient balance between supplies and requirements of crops, to avoid over- and under-fertilization in crop production, and to lower the carbon footprint of crop production.

### Legume–cereal rotations and carbon footprints

Lentil was rotated with wheat in alternate years, a cropping system in which the grain legume was used to partially replace the fallow phase in fallow-wheat or FWW systems or to diversify the wheat monoculture system. This was considered an alternative and more desirable system compared with the traditional rotation systems. On average, wheat in the LentW system produced a similar amount of grain as in the ContW system, averaging 1,860±150 kg ha^−1^ per year, but the former did so with 29% less N fertilizer (Table 2). Consequently, fertilizer N use efficiency for wheat in the LentW system averaged 80% greater than for ContW in dry years, 97% greater in normal years and 36% greater in wet years. Legume–rhizobial associations are known to be an effective solar-driven N_2_-fixing system in which atmospheric N_2_ is transformed into ammonia to provide a large portion of the N requirements for plant growth^31^. A portion of the fixed-N remains in the crop roots, nodules and in the soil rhizodeposits contributing to the N pools in the soil and benefiting subsequent crops^30^,^31^,^45^. Although lentil crops usually have a lower aboveground biomass than wheat crops^30^, the higher N concentration in lentil residues provides greater N-benefits to subsequent crops^46^. It is clear that the use of grain legumes to replace the summerfallow phase of the rotation is one of the key components for obtaining a reduced or negative carbon footprint in wheat cropping.

### Sensitivity of carbon footprints to key contributing factors

An important question is how sensitive is the carbon footprint value to the key contributing factors. To assess this, we conduct three sensitivity tests: one is to test how sensitive the carbon footprint values are in response to the gain or loss of soil organic carbon over time; the second is to determine the sensitivity of N_2_O emission profiles and the resulting carbon footprints to the methods of N fertilizer application; and the third is to test whether the carbon footprint values differ with the calculations at the 100- versus 20-year timeframes of global warming potential for N_2_O. These sensitivity tests may provide some insights into the usefulness of the results obtained from this long-term experiment to other regions with environmental conditions similar to those on the North American prairies. Also, these tests may shed some light on the longer- versus shorter-term benefits of the net C sequestering farming practices for policy relevance.

**Figure 5:**
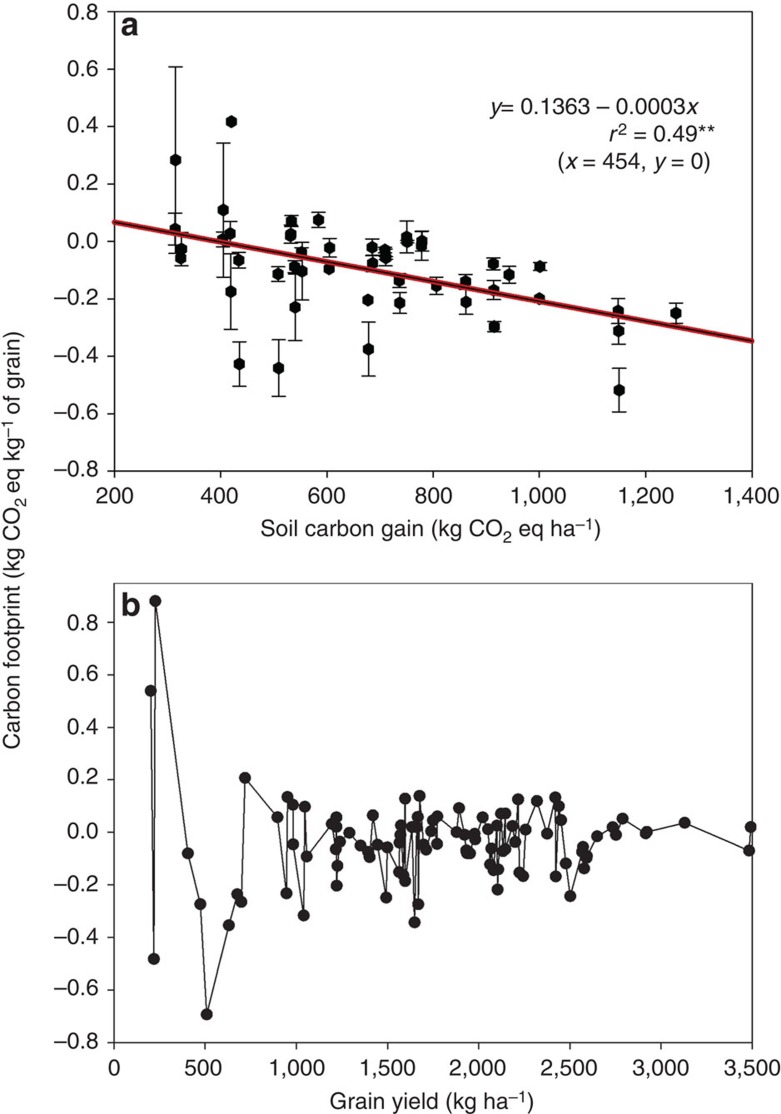
The relationship between carbon footprint and soil carbon gain and crop yield in wheat. (**a**) Wheat carbon footprint (with the line bars at the data point being the standard errors) is equal to zero at soil carbon gain of 454 kg CO_2_ eq ha^−1^ and it becomes negative (more desirable) with soil carbon gain greater than 454 kg CO_2_ eq ha^−1^; and (**b**) wheat carbon footprint values do not have a clear correlation with the grain yields, even though grain yields serve as the denominator in the calculation of carbon footprint values, suggesting that the carbon footprint of wheat production is an outcome of a complex of various factors.

In the first sensitivity test, we found that the gain in SOC over time played a significant role in offsetting carbon emissions ascribed to crop inputs and ultimately impacting the carbon footprint values in this study (Fig. 2, Table 3). Wheat carbon footprint was a linear function of the quantity of SOC gains, with each kg of soil carbon gain lowering the carbon footprint values by 0.0003 U (Fig. 5a). During the 25-year period, the soils under these four rotation systems gained SOC equivalent to an amount of 787 CO_2_ eq ha^−1^ per year (ranging from 310 to 1,280 CO_2_ eq ha^−1^ per year). The sensitivity test reveals that a gain of SOC at an amount of 454 CO_2_ eq ha^−1^ per year or more can result in negative carbon footprints. In other words, a 42% decrease of SOC gain from the current level of the gain measured in the long-term experiment (that is, decrease the SOC gain from 787 to 454 CO_2_ eq ha^−1^ per year) would be the ‘breakeven’ point (that is, the production of wheat becomes carbon neutral). Conversely, with a hypothetic increase of SOC gain by another 25% from the current level of gain (that is, from the current average gain of 787 to a hypothetic gain of 983 CO_2_ eq ha^−1^ per year), the carbon footprint value would become 77% more negative than those estimated in the present study. Noteworthy is that the carbon footprint values did not have a clear correlation with grain yields (Fig. 5b), even though grain yields served as the denominator in the calculation of carbon footprint values. This suggests that the carbon footprint of wheat production is an outcome of a complex of various factors, with SOC serving as one of them (more discussion on the effect of SOC on carbon footprints is provided in the Discussion section below).

Wheat crops were fertilized on the basis of annual soil tests, with the annual N fertilizer rate varying from 3.7 to 69 kg N ha^−1^; this is equivalent to an average N fertilizer rate of 44.1 kg N ha^−1^ per year. In the second sensitivity test, we chose two of the crop rotation systems to illustrate whether the methods of N fertilization would have had an impact on carbon emission profiles and the carbon footprints (Table 3). For the legume–cereal rotation, the sensitivity test showed that the N_2_O emission was 116 kg CO_2_ eq ha^−1^ with N fertilizer application based on annual soil tests, whereas the N_2_O emission value was increased to 158 kg CO_2_ eq ha^−1^ if a blanket rate of 44.1 kg N per ha had been otherwise used without soil tests. The former approach reduced N_2_O emissions by 27% annually compared with the latter. As a result, the soil-testing approach lowered the annual carbon footprint of wheat by an average of 7%. Similarly, when the two different N fertilization approaches were compared for the wheat monoculture system (that is, ContW system), the soil-testing approach reduced the N_2_O emissions by an average of 13% as compared with a blanket N application. Between the two crop rotation systems (with the soil-testing approach), the LentW system decreased the N_2_O emissions by an average of 16%, reduced total emissions by 127% and lowered the carbon footprints by 150%, compared with the wheat monoculture system. These results clearly indicate (i) the benefits of soil testing for crop fertilization over a blanket rate of fertilization and (ii) the benefits of using legume–cereal rotation over wheat monoculture systems, in reducing carbon emissions and lowering carbon footprints.

The library parameters and impact assessment used in the carbon footprint calculation in the scientific communities are primarily based on the international standards of 1 kg of N_2_O having the global warming potential of 298 CO_2_ equivalents with a 100-year timeframe^47^. However, to understand whether the N_2_O emissions and the net C sequestering found in this study might differ with the change of the timeframe of global warming potential for N_2_O, we conducted the third sensitivity test to determine the footprint sensitivity in response to the 100- versus 20-year timeframes (Table 3). We found that the effect of the global warming potential timeframes on N_2_O emissions was interacted with water availability. Under drier conditions, timeframe had no effect on N_2_O emissions or the carbon footprint. Under normal and wetter conditions, however, the results from the two timeframes differed significantly. The estimates from a 20-year timeframe decreased N_2_O emissions by 7.9%, leading to 8.6% more negative for the net emissions and 8.3% more negative for the per-yield carbon footprint values, as compared with the 100-year timeframe.

## Discussion

Increasing awareness of climate change and energy security is spurring greater investigation into how farming systems can be better managed to produce high-quality and affordable food in sufficient quantities while minimizing potentially negative impacts on the environment^1^,^3^,^5^. The present study demonstrates that each individual farming practice has its own role in affecting crop yield, but that packaging those individually proven farming practices together in a systematic manner can enable an increase in crop yield, while concurrently reducing the carbon footprint of crop production. In the long-term field experiment, we identified and packaged a suite of improved farming practices for spring wheat grown in semiarid rainfed environments, including (i) the choice of crop rotation system, (ii) the use of soil tests to determine soil residual N and to estimate mineralized N from soil organic matter that may be available for the crops, (iii) the application of fertilizers to balance the nutrient supplies and plant requirements and (iv) the use of grain legumes to replace the fallow phase of the commonly used crop rotations or to diversify the wheat monoculture system. The results of the 25 years of field tests clearly show that wheat crops can be produced with a small or negative carbon footprint if some key farming practices are identified and packaged together at a system level.

Our findings are strongly supported by other studies where improved farming practices increase crop yields without increasing, or even decreasing, carbon emissions. For example, in an UK study, the use of fungicides increased wheat grain yield by 1.78 t ha^−1^ and reduced greenhouse gas emissions from 386 to 327 kg CO_2_ eq per tonne of grain or by 15% (ref. 48). In Denmark, with optimized fertilization and improved agronomic practices, winter wheat increased grain yield by 7.9%, whereas greenhouse gas emissions during the production was reduced by 1.7 g CO_2_ eq MJ^−1^ of ethanol produced^49^. In Poland, the use of fertilizers selectively from low-carbon emitting manufacturers in wheat production significantly increased the sequestration of soil organic carbon, and reduced carbon emissions by 50% (ref. 50). In New South Wales, Australia, wheat had a carbon footprint value of 0.20 kg CO_2_ eq per kg of grain on a production level of 3.5 t ha^−1^, and the footprint value was reduced to 0.15 kg CO_2_ eq per kg of grain when the production level was increased to 5.0 t ha^−1^, or a 25% reduction of the C emissions with the increased yield level^40^.

Our findings are in contrast with many published studies where the production of wheat typically results in positive (undesirable) carbon footprints^40^,^49^,^51^,^52^. In some of those studies, CO_2_ emissions from crop inputs were substantially greater than was sequestered from the atmosphere^51^,^52^ or sub-optimal farming practices were used (for example, over-fertilizing crops, excessive losses of N that was unused by the crop), or the various farming practices used were not integrated together effectively. In some studies, soil carbon changes were not assessed in their carbon footprint calculations^40^,^49^. In a few cases, an incomplete ‘life cycle assessment’ approach was employed and most importantly the carbon sequestration from the atmosphere back to the soil, a critical component in carbon footprint estimates, was not determined.

In the present study, the choice of cropping systems was a key factor influencing wheat carbon footprints. Use of lentil (a grain legume) to replace the summerfallow phase of the wheat-wheat-fallow system reduced the wheat carbon footprint significantly. Based on the fertilizer use in the LentW system (averaging 31.3 kg N ha^−1^) versus that of wheat monoculture (averaging 44.1 kg N ha^−1^), we estimated that approximately 0.14 million tonnes of N fertilizer could be eliminated each year on the Canadian prairie if half of the wheat production areas were established in a legume–cereal rotation system. This switch would lead to an annual reduction of 1.1 million tonnes of greenhouse gas emissions ascribable to crop inputs. This finding does not suggest that any one particular rotation system can be applicable under all situations; however, it is clear that developing and adopting more effective and efficient cropping systems has a key role to play in increasing crop yields while mitigating a significant amount of greenhouse gases in agriculture.

Next was the change of soil organic carbon over time that played a critical role in offsetting carbon emissions associated with crop production inputs. It is well known that the status of carbon in the soil can be influenced by many factors^53^, such as soil texture, environmental conditions, crop management practices and initial soil C level. The traditional cropping systems used on the semiarid prairies for decades before the initiation of this long-term experiment were mainly fallow-wheat or FWW with little N fertilizer added to the system. The base value of SOC measured in 1979 when the LentW system was first introduced in the study was about 33 Mg C ha^−1^ in the 0–15 cm depth. After the 25 years of the experiment, the SOC level at the field has increased, but it is still substantially lower than they were before the land was cultivated. The conversion of native prairies to agricultural lands has resulted in significant loss of SOC in the soil profile, ultimately leading to the decline of soil fertility in the low-productivity semiarid agroecosystem^54^. It is unknown whether the SOC at the experimental site can be enhanced from the current level, but we speculate that a theoretical potential would be to return the SOC to the level near when the soil was broken for agriculture decades ago. Many recent studies have shown that the change of cropping systems from the traditional frequent fallowing to continuous cropping provides greater carbon input to the soil^54^; the increased N fertilization also increases crop productivity and the quantity of carbon in the soil^55^. Further, a large portion of plant residue carbon can be sequestered in more stable soil organo-mineral complexes^56^.

However, the quantity of carbon in the soil can decline over time under unfavourable farming practices such as frequent summerfallowing with intensive tillage^18^, low crop yields^27^,^57^ with limited amounts of crop residues returned to the soil^26^ or use of sub-optimal farming practices^58^. In the present study, two to five tillage operations were used for weed control during the post-harvest fallow period; this may have had negative impacts on soil carbon sequestration as the practice of tilling the soil accelerates the loss of soil carbon^28^,^57^. Some other studies, however, have shown that tillage can influence mineralizable C and microbial biomass^18^, but these changes in mineralizable C do not usually affect crop yields in semiarid climates^44^. It appears that the effect of tillage operation on SOC changes may require a much longer period of time (perhaps several decades^18^) to become distinguishable in semiarid climates.

Putting all these together, we acknowledge that the gain in SOC obtained in this long-term experiment may not be indefinite as the SOC accrual may start reaching a new steady state, as evidenced by the fact that the rate of SOC gain in the last decade (that is, the 2000s) has been smaller compared with the earlier decades. Also, the effect of cropping systems on the SOC gain may approach a new equilibrium^55^. Thus, the future implications of SOC on wheat carbon footprints and thus climatic impacts can be complex. If the last decade is really the new normal, the potential climate impact of SOC gain associated with wheat production systems can be small or slightly negative. With many unknown factors involved in this complex issue, we speculate that three possibilities may occur in the future: (i) the carbon footprints may continue to become more negative, as more advanced technologies are adopted in farming practices, with lower-carbon crop inputs being manufactured and used for crop production; (ii) the per-yield carbon footprints may approach zero, with carbon exports equaling carbon imports in wheat production systems, and there may be no net sequestration of carbon or (iii) the per-yield carbon footprint values may trend to be positive, with the slow erosion of the carbon sequestered in the soil with no new carbon sequestration occurring.

Many process-based models have been employed to characterize the trajectories of past crop yields^24^, and to identify possible strategies to continue increasing crop yields^25^ or for closing the yield gaps between the current level and the potential^6^. The added feature from the actual field measurements of our long-term experiment is that one can (i) assess the effect of adopting improved farming practices on crop productivity and their environmental consequences under the highly-variable semiarid climate; (ii) perform a reasonably complete life cycle assessment of wheat production with robust estimates of the carbon footprints and (iii) understand the potential of converting solar radiation to plant biomass and to offset the carbon emissions from the cultivation of natural carbon-rich ecosystems. The production of large-scale staple crops like wheat has been shown to have significant climate consequences. The findings from this well-managed, replicated, long-term field experiment provide strong evidence that spring wheat can be a net carbon-sequestering crop with a small or negative carbon footprint, if the crop is produced with the integration of proven farming practices. We do not suggest that the net-sequestering goal can be achieved for wheat crops grown in environments different from the semiarid North American prairies, but we do suggest that the production of large-scale staple food crops with advanced farming technologies and practices can increase crop yield, enhance soil carbon sequestration and allow the capture of large society-environmental benefits and other potential co-benefits for human health^59^.

## Methods

### Field plot layout and management

The ContW system had a wheat crop every year; LentW had a wheat crop alternated with a lentil crop every other year; FWW had two wheat crops and summerfallow every 3 years; whereas FFlxW had one wheat crop, one flax crop and summerfallow every 3 years. Agricultural researchers refer to these as ‘rotation phases’. So, the ContW system had one phase (that is, wheat every year); LentW had two phases, FWW had three phases and FFlxW had three phases. All these phases were present each year and were randomized in each replicate. Using 2007 as an example, the field plot layout map shows how the various rotations and their phases were randomized in each of the three replicates (Table 5). For example, FWW system had three plots in each replicate: one plot was for phase-1 (**F**WW), a second plot for phase-2 (F**W**W) and a third plot for phase-3 (FW**W**). Thus, for the four rotation systems, there were a total of nine rotation phases that were randomized within each of the three replicates, making a total of 27 plots each year (9 plots per replicate × 3 replicates). All rotations were cycled over the years on their corresponding plots. Each plot was 10 m by 20 m in size.

In each year, soil NO_3_-N (0–0.6 m depth) and soil P (0–0.15 m depth) levels were measured in each plot in fall just before freeze up and these values, along with nutrient application guidelines provided by the Soil Testing Laboratory of the University of Saskatchewan, were used to determine fertilizer rates to be applied in the spring to the following crop. The crops received in-crop weed control as needed using recommended herbicides at label rates. In the fall, 2,4-D was applied to all plots to control winter annual weeds. Plots being planted generally received one preseeding tillage operation with a heavy-duty sweep cultivator and mounted harrow to prepare the seedbed, whereas plots being summerfallowed received two to five tillage operations with a cultivator or rod weeder to control weeds. Full-sized farm equipment was used for all cultural and tillage operations. All the other cropping practices such as planting and harvesting were those recommended for crop production in the local area.

### Data collection

At full maturity, an area of 2.32 m^2^ was hand-harvested in each plot for grain and straw biomass, and the six central rows of plants in each plot were combine-harvested for grain yield. The N concentrations in grain and straw were measured using the standard micro-Kjeldahl method. Root dry weight was estimated using the published model^30^ where root biomass was taken as a proportion of straw biomass, varying with growing conditions. All crop inputs such as seeds, fertilizers, pesticides, fossil fuels and so on were recorded for each of the rotation systems.

Soil organic carbon measured in 1979 (when the LentW rotation was first included in the systems) was used as the starting value or baseline, with subsequent SOC measurements taken in 1981, 1985, 1990, 1993, 1996, 1999, 2003 and 2009 (ref. 55). At each measurement, two soil samples were taken at random within the central part of each plot. The soils were sampled using soil cores with a 5-cm diameter probe, and the cores were then divided into 0–15 and 15–30 cm depths. The two cores were bulked within each depth, air dried and sieved (<2 mm). Representative subsamples of the <2 mm soil were ground with a roller mill (<153 μm) and a 20-mg subsample analysed for organic C with an automated combustion technique (Carlo Erba, Milan, Italy). The organic C was obtained after pre-treatment of the soil with phosphoric acid to remove inorganic C. The SOC concentrations were converted to weight per volume using soil bulk densities. Annualized SOC gains or losses for each cropping system were calculated using the difference in SOC value at two consecutive measurement dates divided by the number of years between samplings.

Sampling depth is a complex factor. In the semiarid prairies where tillage is usually shallow (no more than 15–20 cm), most of the SOC changes occur near the soil surface. Thus, sampling usually is in increments only to the 15- or 30-cm soil depth. Typically, absolute differences in SOC among treatments increase with the depth sampled, but statistical significance declines because total mass of C measured and its variability increase more than the difference among cropping treatments^60^. In fact, significant differences are often observed only in the surface 10 cm depth, if at all^27^,^61^. We have therefore confined our discussion on SOC primarily to the changes of SOC in the 0- to 15-cm soil depth in this experiment. Some more detailed explanations and discussions have been given in previous publications^55^,^61^ that specifically deal with soil carbon changes found in this long-term field experiment.

### Emission estimate and carbon footprint calculation

In the estimate of greenhouse gas emissions, a boundary was set from the manufacture, transportation, storage and delivery of crop inputs (for example, fertilizers and pesticides) to harvest crops^51^. Within the set boundaries, a country-specific approach^51^,^62^ was used to estimate greenhouse gas emissions from all sources, with site-measured data from the semiarid North American prairies^22^,^45^ coupled with empirical modelling^62^. The emissions from energy use and N_2_O emissions from non-energy sources include those from (i) crop residue decomposition, (ii) inorganic N and phosphorus fertilizer application, (iii) N losses from leaching and volatilization, (iv) application of herbicides, fungicides and insecticides, (v) fuel used in various farming operations and (vi) fossil energy used in the processes of manufacture, transportation, storage and delivery of fertilizers and pesticides to the farm gate. The emissions of N_2_O and CH_4_ were converted into CO_2_ eq^40^,^51^,^52^, which allows the comparison of different cropping systems using the same functional unit.

When a crop is harvested, straw and roots are left in the field to decompose. These crop residues become an important N source for nitrification and denitrification, contributing directly and indirectly to N_2_O emissions^20^,^63^. The amount of N_2_O contributed by the decomposition of crop straw and roots is directly related to their N concentration and biomass yield^20^, thus, straw and root N concentrations have been incorporated in the emission estimates. The quantity of crop residue N was obtained using the aboveground and belowground crop residue biomass multiplied by their respective N concentrations. Thus, N_2_O emissions from farmland are the collective result of N that has entered the soil from inorganic fertilizers, the decomposition of crop residues, volatilization of NH_3_ and nitrate losses via leaching and denitrification in wet conditions.

A country-specific model for the determination of N_2_O emission factors for Canadian conditions has been developed and published^62^; this model is primarily based on the actual measurements taken from this Swift Current long-term experiment, along with other related studies^22^,^45^. Direct emissions from crop residue decomposition, fertilizer N application and the fraction of N that is subject to leaching are found to be a function of the ratio of Pr to potential PE:


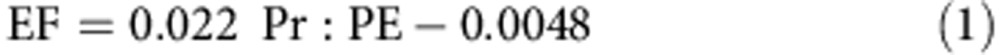



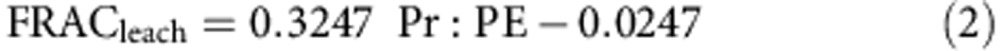


where, EF is the emission factor with a unit of kg N_2_O-N kg^−1^ of N; Pr/PE is the ratio of Pr to PE during the growing season and FRAC_leach_ is the fraction of fertilizer- and crop residue-N subject to leaching.

Emissions of N_2_O from inorganic N fertilizer applications and crop residue decomposition are then estimated as:


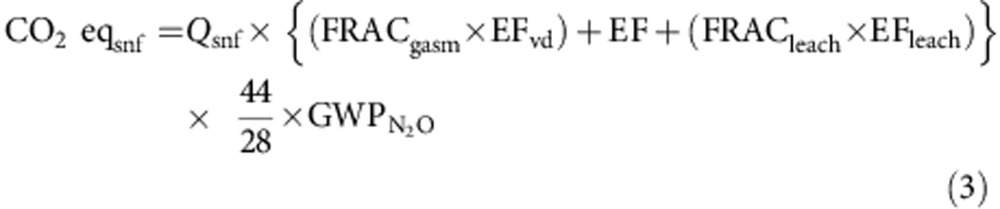



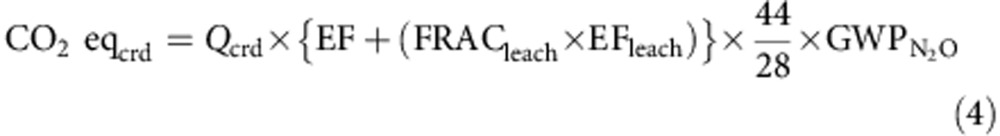


where, CO_2_eq_snf_ and CO_2_eq_crd_ are total emissions from the inorganic N fertilizer application and crop residue decomposition (kg CO_2_ eq ha^−1^), respectively; *Q*_snf_ is the quantity of synthetic N fertilizer applied (kg N ha^−1^); *Q*_crd_ is the quantity of crop residue N; FRAC_gasm_ is the fraction of inorganic fertilizer-N that volatilized as NH_3_- and NO_x_-N (FRAC_gasm_=0.1 kg N kg^−1^ N); EF_vd_ is the N_2_O emission factor for volatilized NH_3_- and NO_x_-N (EF_vd_=0.01 kg N_2_O-N kg^−1^ N); EF_leach_ is the N_2_O emission factor for nitrate leaching (EF_leach_=0.0075, kg N_2_O-N kg^−1^ N); 44/28 is the conversion coefficient from N_2_O-N to N_2_O and the GWP_N2O_=298 for the global warming potential of N_2_O for the 100-year timeframe and the GWP_N2O_=268 for the 20-year timeframe^64^.

In our presentation, the calculations of carbon footprint are primarily based on the global warming potential in a time horizon of 100 years as this is the recommendation by the IPCC and is used by regulators^47^. However, to assess the potential usefulness for overall policy relevance, we also calculated the carbon footprint values using GWP_N2O_=268 for the 20-year timeframe in converting N_2_O to CO_2_ equivalents. The comparison between the two sets of calculations may give some indications about the sensitivity of the results to the choice of timeframe and help us to understand the shorter- versus longer-term benefits. This effort is based on the understanding that global warming potential is a relative measure of how much heat a greenhouse gas traps in the atmosphere^65^.

Although having all N_2_O emission estimates based on field measurements would be ideal, such long-term data sets do not exist. The estimates in the present study were based on a well-scrutinized method^62^ that utilized measured data from the long-term experiment and other relevant studies. The emission estimates in the present study compared well to those field-measurements from the specific rotation systems discussed previously^22^,^45^, and those from similar studies of comparable systems^66^.

Herbicides, fungicides and insecticides were used as needed and at recommended rates in the experiment. In the emission estimates, we used average emission factors of 23.2 kg CO_2_ eq kg^−1^ of active ingredient for herbicides and 13.8 kg CO_2_ eq kg^−1^ of active ingredient for fungicides and insecticides. These emission factors were based on the estimates of fossil energy use during the processes of manufacture, transportation, storage and delivery of the products to the farm gate. The total emissions associated with the use of pesticides were calculated by multiplying the emission factors with the quantity of the pesticides actually applied to the crops in each rotation system. The resulting values are in the range published by others from the North American prairies^22^,^51^,^67^, and areas of the semiarid Australia with production systems similar to ours^40^. Although the true emission values for individual pesticides may differ from those calculated using our constant factors, the relative values are believed to be reasonable given that the portion of the overall carbon footprint contributed by pesticides under dryland conditions is generally small^37^,^48^,^51^.

The emissions associated with miscellaneous farming operations such as planting, applying pesticides and harvesting were estimated using a factor of 14 kg CO_2_ eq ha^−1^ for planting, 5 kg CO_2_ eq ha^−1^ for pesticide application and 37 kg CO_2_ eq ha^−1^ for harvesting. These emission factors are also in the range of the values published by others for the North American prairies^22^,^36^,^37^,^68^.

Finally, summerfallow is a farming practice typically used in the arid and semiarid prairies to control weeds through tillage and to conserve soil moisture by leaving the land unplanted for an entire growing season. The N_2_O emissions during the summerfallow period were estimated based on the actual inputs (such as fuel use in tillage operations) plus indirect emissions from non-input sources such as residual soil N and the decomposition of organic matter present in the soil^62^; the latter portion was to account for these emissions not captured by the input-based method^22^,^45^.

### Statistical analysis

To avoid the confounding effect of highly variable growing season precipitation on the treatments, we categorized the 25 (1985–2009) experimental years into three water-availability conditions: dry, normal and wet (Table 4). In 7 dry years, growing season (1 May to 31 August) Pr averaged 186 mm, PE 641 mm (Pr to PE ratio=0.291) and mean air temperature 14.2 °C. In 5 wet years, the Pr averaged 383 mm, PE 559 mm (Pr/PE=0.687) and mean air temperature 12.7 °C. The remaining 13 years had weather conditions near the long-term means, with Pr averaging 286 mm, PE 582 mm (Pr/PE=0.496) and air temperature 13.3 °C.

All phases of each rotation were present every year and randomized in each replicate; this allowed the analysis to be performed on a complete rotation basis. We determined all variables (for example, production inputs, crop yields, C emissions and so on) for the complete rotation systems (that is, all phases in each year) and then converted the results into annualized values. For example, the annualized grain yield for the FWW system in replicate I in 2007 (Table 5) was the sum of the yields from three plots (plot #3, #5, #8) divided by 3 (zero yield in plot #5, the fallow phase). Thus, the individual years were considered independent in the statistical analyses. There were 7 dry years, 13 normal years and 5 wet years, with three replicates in each year; the mixed effect model gives the best results for the least significant differences between the four rotation systems, with random effects being: 3 × 7, 3 × 13 and 3 × 5 in dry, normal and wet years, respectively^69^.

Preliminary analysis revealed significant interactions between cropping systems and water-availability categories (dry, normal and wet) for most of the variables assessed, therefore, treatment effects were primarily discussed for each of the three water-availability categories. Means and standard errors of the means were estimated on the basis of three replicates per year × the number of years in each water-availability category (*n*=3 × 7, 3 × 13 and 3 × 5 in dry, normal and wet years, respectively). Regressions were used to determine the relationships between grain yield and precipitation, between N input and greenhouse gas emissions, between N input and N uptake by plants, and between N input and N-surplus. An analysis of covariance was used to determine the relative contribution of various crop input factors to the carbon footprint values^69^,^70^.

Finally, sensitivity tests were conducted to assess how sensitive the N_2_O emission profiles and the resulting carbon footprints are to (i) the methods of N fertilizer application; (ii) the level of SOC gain or loss over years and (iii) the estimates of carbon footprints based on the 100- versus 20-year timeframes of global warming potential.

## Author contributions

Y.G., C.L. and Q.C. conceived the study and constructed the models; Q.C and Y.G. performed the statistical analyses; R.L.L., C.A.C. and R.P.Z are involved in field data collection; all authors contributed to writing the paper.

## Additional information

**How to cite this article:** Gan, Y. *et al.* Improving farming practices reduce the carbon footprint of spring wheat production. *Nat. Commun.* 5:5012 doi: 10.1038/ncomms6012 (2014).

## Supplementary Material

Supplementary Tables Supplementary Tables 1-2

## Figures and Tables

**Table 1:** Carbon footprints of spring wheat on the basis of per area and per unit of grain yield.

Cropping system*	**Per-area carbon footprint**				**Per-yield carbon footprint**			
	**Dry**†	**Normal**†	**Wet**†	**Mean**	**Dry**	**Normal**	**Wet**	**Mean**
	**kg CO_2_ eq ha^−1^ per year**				**kg CO_2_ eq kg^−1^ of grain per year**			
FFlxW	−29	−98	−16	−62	−0.003	−0.049	−0.005	−0.027
FWW	−116	−254	−233	−218	−0.168	−0.166	−0.109	−0.164
ContW	−137	−304	−265	−243	−0.148	−0.167	−0.154	−0.151
LentW	−379	−634	−580	−552	−0.570	−0.322	−0.249	−0.377
LS-mean	−165	−323	−274	−256	−0.223	−0.176	−0.129	−0.146
LSD (0.05)‡	71	53	117	44	0.256	0.048	0.059	0.079
*P*-value	<0.01	<0.01	<0.01	<0.01	0.02	<0.01	<0.01	<0.01

LS-mean, least-square mean; LSD, least significant difference.

^*^The four rotation systems are: (i) fallow-flax (*Linum usitatissimum*)-wheat (FFlxW), (ii) fallow-wheat-wheat (FWW), (iii) continuous wheat (ContW) and (iv) lentil (*Lens culinaris* Medikus)-wheat (LentW).

^†^Dry years with annual precipitation (Pr) below 210 mm and the ratio of Pr to evapotranspiration (Pr/PE)=0.291; normal years with annual Pr between 211 and 340 mm and Pr/PE=0.495 and wet years with annual Pr between 341 and 420 mm and Pr/PE=0.687.

^‡^LSD between the four rotation systems determined using mixed effect model (*n*=3 × 7, 3 × 13 and 3 × 5 in dry, normal and wet years, respectively).

**Table 2:** Wheat grain yield and its relation to fertilizer-N input and NUE.

**Water availability**	Cropping system*	**Grain yield**			**N fertilizer**	**NUE**
		**Mean**†	**Min**	**Max**	**Mean**†	**Mean**†
		**kg ha^−1^**			**kg ha^−1^**	**kg grain kg^−1^ of N**
Dry	FFlxW	1,119	226	1,879	37.3	63.4
	FWW	976	405	1,499	19.6	60.9
	ContW	1,086	201	1,712	38.7	40.8
	LentW	1,021	218	2,063	22.9	73.6
	LSD (0.05)‡	212	—	—	—	37.4
	*P*-value	0.15	—	—	—	0.34
Normal	FFlxW	2,124	952	3,493	46.5	76.6
	FWW	1,604	1,221	2,200	26.2	77.5
	ContW	2,054	982	3,130	45.5	63.9
	LentW	2,180	1,040	3,484	34.1	126.0
	LSD (0.05)‡	205	—	—	—	48.7
	*P*-value	<0.01	—	—	—	0.04
Wet	FFlxW	2,458	2,186	2,923	45.6	61.0
	FWW	1,735	1,402	1,937	24.1	77.0
	ContW	2,248	1,566	2,917	48.1	47.9
	LentW	2,389	2,104	2,594	37.0	65.2
	LSD (0.05)‡	243	—	—	—	27.6
	*P*-value	0.03	—	—	—	0.03
Mean	FFlxW	1,909	226	3,493	43.7	69.8
	FWW	1,455	405	2,200	23.9	72.8
	ContW	1,822	201	3,130	44.1	54.2
	LentW	1,897	218	3,484	31.5	99.1
	LSD (0.05)‡	138	—	—	—	27.3
	*P*-value	<0.01	—	—	—	<0.01

LSD, least significant difference; Max, maximum; Min, minimum; NUE, N use efficiency.

^*^The four rotation systems are: (i) fallow-flax (*Linum usitatissimum*)-wheat (FFlxW), (ii) fallow-wheat-wheat (FWW), (iii) continuous wheat (ContW) and (iv) lentil (*Lens culinaris* Medikus)-wheat (LentW).

^†^Least square means.

^‡^LSDs between the four rotation systems determined using mixed effect model (*n*=3 × 7, 3 × 13 and 3 × 5 in dry, normal and wet years, respectively).

**Table 3:** Sensitivity of carbon footprints to the method of N application and the timeframe of global warming potential.

**Effect**	**N**_2_**O emissions**	**N**_2_**O emissions as % of total emission**	**Net emissions**	**Carbon footprint**
	**kg CO**_2_-eq **ha**^−1^	**%**	**kg CO**_2_-eq **ha**^−1^	**kg CO**_2_-eq **kg**^−1^ **of grain**
*Sensitivity to N fertilizer application methods*				
*Wheat rotating with a grain legume*				
Soil test rate	116	20.6	−552	−0.377
Blanket	158	28.8	−510	−0.351
s.e.m.†	16**	1.9**	30**	0.052**
*Wheat in monoculture*				
Soil test rate	138	24.5	−244	−0.153
Blanket	159	26.1	−243	−0.151
s.e.m.†	18*	2.0 NS	25 NS	0.020 NS
*Sensitivity to the timeframe of global warming potential*				
*Dry*				
100-year	49	13.8	−156	−0.214
20-year	46	13.0	−162	−0.224
s.e.m.‡	4 ns	0.9 NS	30 NS	0.061 NS
*Normal*				
100-year	146	25.2	−315	−0.173
20-year	135	24.1	−336	−0.184
s.e.m.‡	7 NS	0.8 NS	30**	0.018*
*Wet*				
100-year	238	33.9	−269	−0.127
20-year	221	33.0	−303	−0.143
s.e.m.‡	15*	1.1 NS	48**	0.022*

s.e.m., standard error of the mean.

^†^Significances between the two methods of N fertilizer application, across years (*n*=24).

^‡^Significances between the two timeframes, across all treatments and years (*n*=99).

^*^significant at P≤0.05; **significant at P≤0.01; NS, not significant with P≥0.05.

**Table 4:** N_2_O emission and N-leaching factors in relation to weather parameters.

**Year**	Pr (mm)*	**Mean T (°C)**	**PE**†	**Pr/PE**	**N**_2_**O emission factors**	**N-leaching factor (FRAC**_Leach_)
*Dry*						
1985	177	12.6	589	0.2997	0.0018	0.0726
1987	187	14.1	636	0.2933	0.0017	0.0705
1988	215	15.3	700	0.3073	0.0020	0.0751
1990	204	13.7	624	0.3270	0.0024	0.0815
2001	147	14.5	664	0.2215	0.0001	0.0472
2003	209	14.7	641	0.3260	0.0024	0.0812
2007	166	14.4	634	0.2625	0.0010	0.0605
Mean	186	14.2	641	0.291	0.0016	0.069
S.e.m.	9	0.3	13	0.014	0.0003	0.004
*Normal*						
1986	329	13.2	569	0.5781	0.0079	0.1630
1989	332	13.5	569	0.5832	0.0080	0.1647
1992	278	12.2	554	0.5015	0.0062	0.1381
1994	240	14.2	619	0.3884	0.0037	0.1014
1996	318	12.4	545	0.5845	0.0081	0.1651
1997	247	14.0	623	0.3970	0.0039	0.1042
1998	268	14.9	653	0.4110	0.0042	0.1088
1999	276	12.8	546	0.5066	0.0063	0.1398
2000	325	13.5	588	0.5528	0.0074	0.1548
2004	332	11.9	518	0.6411	0.0093	0.1835
2005	269	13.1	562	0.4787	0.0057	0.1307
2006	277	14.0	621	0.4455	0.0050	0.1199
2009	226	12.9	598	0.3783	0.0035	0.0981
Mean	286	13.3	582	0.495	0.0061	0.136
S.e.m.	10	0.2	11	0.024	0.0005	0.007
*Wet*						
1991	364	13.4	589	0.6189	0.0088	0.1763
1993	401	12.2	544	0.7383	0.0114	0.2150
1995	380	12.8	551	0.6899	0.0104	0.1993
2002	420	12.0	531	0.7904	0.0126	0.2319
2008	351	13.3	584	0.6015	0.0084	0.1706
Mean	383	12.7	559	0.687	0.0103	0.198
S.e.m.	12	0.3	11	0.035	0.0008	0.011

Pr, precipitation; PE, potential evapotranspiration.

^*^Growing season (1 May—31 August) Pr and air temperatures are obtained from the weather station at the experimental site.

^†^PE data are provided by Environment Canada.

**Table 5:** Randomization of different cropping systems and their rotation phases in each replicate.

**Replicate**	**Plot #**	**Rotation system**	**Rotation phase**	**Crop**	**Fertilizers (N-P-K-S)**	
					**46-0-0**	**12-51-0-0**
1	1	FFlxW	2	Wheat	90	38
1	2	LentW	2	Lentil	7	38
1	3	FWW	2	Wheat	90	38
1	4	LentW	1	Wheat	113	38
1	5	FWW	1	Fallow	0	0
1	6	FFlxW	1	Fallow	0	0
1	7	ContW	1	Wheat	116	38
1	8	FWW	3	Wheat	130	38
2	9	LentW	1	Wheat	74	38
2	10	FWW	3	Wheat	121	38
2	11	ContW	1	Wheat	107	38
2	12	FFlxW	1	Fallow	0	0
2	13	FWW	1	Fallow	0	0
2	14	FFlxW	2	Wheat	106	38
2	15	LentW	2	Lentil	0	38
2	16	FWW	2	Wheat	80	38
3	17	FFlxW	1	Fallow	0	0
3	18	FWW	1	Fallow	0	0
3	19	ContW	1	Wheat	124	38
3	20	FFlxW	2	Wheat	84	38
3	21	LentW	2	Lentil	0	38
3	22	FWW	2	Wheat	92	38
3	23	LentW	1	Wheat	98	38
3	24	FWW	3	Wheat	121	38

The plot layout is extracted from the 2007 plan, as an example, to illustrate how the rotation phases are randomized in each replicate in each year. The variable rates of fertilizers (kg ha^−1^) are based on soil tests.

The four cropping systems—(i) fallow-flax-wheat (FFlxW), (ii) fallow-wheat-wheat (FWW), (iii) continuous wheat (ContW) and (vi) lentil-wheat (LentW), were tested in the field with each plot being 10 m × 20 m in size.
