# Supplementary material for: Improving farming practices reduces the carbon footprint of spring wheat production
Source: Nat Commun. 2014 Nov 18;5:5012. doi: 10.1038/ncomms6012 (PMC4243251; doi:10.1038/ncomms6012)
Supplement: Supplementary Tables — 1-2 [file ncomms6012-s1.pdf]

Supplementary Table 1 – Summary of covariance analysis for per-area carbon footprint and per-yield carbon footprint for different wheat production systems at Swift Current, Saskatchewan.

Source [a]	Overall, DF=95			Dry, DF=9			Normal, DF=43			Wet, DF=16		
	Sum of squares	%		Sum of squares	%		Sum of squares	%		Sum of squares	%	
**Per-area carbon footprint**												
System (Sy)[b]	9730360	**82.18**	**[c]	1213212	**77.90**	**	6684288	**86.12**	**	2306392	**91.49**	**
Nitrogen (N)	346348	**2.93**	**	324456	**20.83**	**	512903	**6.61**	**	156349	**6.20**	**
N*N	51673	0.44	**	3117	0.20	ns	31617	0.41	**	17220	0.68	**
Yield (Y)	377462	**3.19**	**	9047	0.58	ns	24018	0.31	**	18310	0.73	**
Y*Y	19988	0.17	**	1	0.00	ns	193	0.00	ns	2453	0.10	**
Precip (Pr)	195821	1.65	**	2139	0.14	ns	31884	0.41	**	12339	0.49	**
Pr*Sy	22752	0.19	**	731	0.05	ns	4809	0.06	ns	1264	0.05	*
PrPE	641	0.01	ns	487	0.03	ns	26271	0.34	**	6677	0.26	**
PrPE*Sy	2329	0.02	ns	947	0.06	ns	4457	0.06	ns	13	0.00	ns
Farm op. (F)	123388	1.04	**	32	0.00	ns	22	0.00	ns	0	0.00	.
F*Sy	18250	0.15	**	1423	0.09	ns	22935	0.30	**	0	0.00	ns
Crop res (Rs)	376312	**3.18**	**	749	0.05	ns	404372	**5.21**	**	0	0.00	ns
Rs*Sy	535	0.00	ns	1137	0.07	ns	3443	0.04	ns	0	0.00	ns
Soil C (Sc)	523741	**4.42**	**	0	0.00	.	0	0.00	.	0	0.00	.
Sc*Sy	456	0.00	ns	0	0.00	.	0	0.00	.	0	0.00	.
Y*Sc*Sy	9220	0.08	*	0	0.00	.	503	0.01	ns	0	0.00	.
N*Y*Sy	10095	0.09	**	0	0.00	.	2003	0.03	ns	0	0.00	.
Phosphrous	1129	0.01	ns	0	0.00	.	1422	0.02	ns	0	0.00	.
Pest	2382	0.02	ns	0	0.00	.	1292	0.02	ns	0	0.00	ns
Residual	27486	0	.	0	0	.	5454	0	.	0	0	.
**Per-area carbon footprint**												
System (Sy)[b]	4.003	**61.20**	**	2.450	**60.42**	**	1.586	**77.32**	**	0.384	**88.04**	**
Nitrogen (N)	0.408	**6.23**	**	0.374	**9.22**	**	0.092	**4.50**	**	0.028	**6.32**	**
N*N	0.082	1.25	**	0.100	2.46	**	0.017	0.83	**	0.003	0.65	**
Yield (Y)	0.954	**14.59**	**	0.825	**20.35**	**	0.169	**8.24**	**	0.016	**3.61**	**
Y*Y	0.068	1.04	**	0.036	0.88	*	0.016	0.79	**	0.002	0.36	**
Precip (Pr)	0.033	0.50	**	0.010	0.24	*	0.008	0.40	**	0.002	0.57	**
Pr*Sy	0.038	0.59	*	0.157	3.88	**	0.002	0.10	*	0.000	0.10	*
PrPE	0.004	0.06	ns	0.016	0.40	*	0.008	0.40	**	0.002	0.36	**
PrPE*Sy	0.008	0.12	ns	0.024	0.59	*	0.005	0.24	**	0.000	0.00	ns
Farm OP. (F)	0.016	0.25	ns	0.004	0.10	ns	0.000	0.00	ns	0.000	0.00	.
F*Sy	0.001	0.02	ns	0.059	1.46	*	0.005	0.24	**	0.000	0.00	ns
Crop res (Rs)	0.098	1.50	**	0.001	0.02	ns	0.124	**6.05**	**	0.000	0.00	ns
Rs*Sy	0.006	0.09	ns	0.000	0.00	ns	0.006	0.31	**	0.000	0.00	ns
Soil C (Sc)	0.193	**2.95**	**	0.000	0.00	.	0.000	0.00	.	0.000	0.00	.
Sc*Sy	0.065	1.00	*	0.000	0.00	.	0.000	0.00	.	0.000	0.00	.
Y*Sc*Sy	0.181	2.77	**	0.000	0.00	.	0.009	0.42	**	0.000	0.00	.
N*Y*Sy	0.120	1.83	**	0.000	0.00	.	0.001	0.07	*	0.000	0.00	.
Phosphrous	0.017	0.26	ns	0.000	0.00	.	0.000	0.02	ns	0.000	0.00	.
Pest	0.002	0.04	ns	0.000	0.00	.	0.001	0.03	ns	0.000	0.00	ns
Residual	0.002	0.04	ns	0.000	0.00	.	0.001	0.03	ns	0.000	0.00	ns

[a]Sources of variance include nitrogen fertilizer (N), grain yield (Y), growing-season precipitation (Pr), the ratio of precipitation to evapotranspiration (PrEP), various on-farm cultural and tillage operations (F), crop residues (Rs), soil organic carbon gain (Sc), phosphorus fertilizer and pesticides; [b]The four cropping systems evaluated are fallow-flax-wheat, fallow-wheat-wheat, continuous wheat, and lentil-wheat; [c] **,*, ns, denote significance at $P \leq 0.01$, $P \leq 0.05$, and not significant, respectively.

Supplementary Table 2 - Percentages of greenhouse gas emissions from various crop inputs for spring wheat grown under dry- (7-yrs), normal- (13-yrs), and wet- (5-yrs) conditions during the 1985-2009 period at Swift Current, Saskatchewan, Canada

Water availability	Cropping system[a]	N-fert. manufacture[b]	N-fert. application	P-fertilization	Pesticides	Farming operation	Crop residues	Fallow effect
		%						
Dry	FFlxW	39.6 (7.0)[c]	11.5 (2.1)	4.8 (1.1)	10.0 (2.4)	31.3 (5.2)	2.9 (0.6)	0.0 (0.0)
	FWW	27.4 (2.2)	8.1 (0.8)	3.3 (0.5)	8.1 (1.3)	31.3 (3.4)	3.1 (0.8)	18.8 (5.0)
	ContW	42.9 (5.2)	12.4 (1.4)	4.1 (0.6)	8.6 (1.4)	28.7 (3.5)	3.1 (0.9)	0.0 (0.0)
	LentW	33.1 (5.5)	9.4 (1.4)	5.6 (0.9)	11.3 (1.9)	35.4 (3.9)	5.2 (1.5)	0.0 (0.0)
Normal	FFlxW	33.7 (3.0)	24.4 (2.4)	2.8 (0.4)	5.9 (0.9)	18.6 (1.2)	14.7 (4.0)	0.0 (0.0)
	FWW	24.5 (2.0)	17.9 (1.7)	2.3 (0.2)	5.1 (0.6)	22.7 (1.8)	12.6 (2.8)	14.9 (1.6)
	ContW	33.2 (3.0)	24.4 (2.6)	2.5 (0.3)	5.5 (0.7)	19.2 (1.6)	15.2 (4.2)	0.0 (0.0)
	LentW	28.5 (3.4)	20.4 (2.5)	3.2 (0.3)	6.3 (0.9)	19.7 (1.8)	21.8 (4.4)	0.0 (0.0)
Wet	FFlxW	28.3 (2.5)	35.6 (3.6)	2.2 (0.2)	3.8 (0.5)	15.5 (1.3)	14.6 (4.7)	0.0 (0.0)
	FWW	21.0 (1.1)	26.7 (2.9)	2.1 (0.2)	3.9 (0.5)	19.9 (1.5)	12.7 (2.3)	13.9 (4.4)
	ContW	30.0 (2.0)	37.2 (1.1)	2.2 (0.1)	3.3 (0.4)	15.1 (0.5)	12.2 (2.8)	0.0 (0.0)
	LentW	26.5 (2.0)	32.8 (1.2)	2.5 (0.2)	3.7 (0.4)	16.0 (0.8)	18.5 (2.9)	0.0 (0.0)
Mean	FFlxW	34.2 (2.6)	23.0 (2.3)	3.2 (0.4)	6.6 (0.9)	21.5 (2.0)	11.3 (2.5)	0.0 (0.0)
	FWW	24.6 (1.3)	16.9 (1.7)	2.5 (0.2)	5.7 (0.6)	24.5 (1.6)	9.9 (1.7)	15.8 (1.8)
	ContW	35.3 (2.3)	23.6 (2.2)	2.9 (0.3)	5.9 (0.6)	21.1 (1.6)	11.2 (2.4)	0.0 (0.0)
	LentW	29.4 (2.3)	19.8 (2.1)	3.8 (0.4)	7.2 (0.9)	23.4 (2.1)	16.5 (2.8)	0.0 (0.0)

[a] The four rotation systems are: (1) fallow-flax (*Linum usitatissimum*)-wheat (FFlxW), (2) fallow-wheat-wheat (FWW), (3) continuous wheat (ContW), and (4) lentil (*Lens culinaris* Medikus)-wheat (LentW); all receiving fertilizers based on soil test recommendation.

[b] Includes emissions from manufacture, transportation, storage, and delivery of products to the farm gate.

[c] The numbers in parentheses are one standard error of the means (*n* = 7, 13, and 5 in dry, normal, and wet years, respectively).
